# Efficacy of Chinese Herbal Injections for Elderly Patients With pneumonia—A Bayesian Network Meta-analysis of Randomized Control Trials

**DOI:** 10.3389/fphar.2021.610745

**Published:** 2021-05-21

**Authors:** Yang Yuan, Quan Zheng, Zhilin Si, Juhua Liu, Zhi Li, Lian Xiong, Pan Liu, Xu Li, Chengshi He, Jinghong Liang

**Affiliations:** ^1^ Hospital of Chengdu University of Traditional Chinese Medicine, Chengdu, China; ^2^ The First People’s Hospital of Suining City, Suining, China; ^3^ Chengdu Second People’s Hospital, Chengdu, China; ^4^ Department of Maternal and Child Health, School of Public Health, Sun Yat-sen University, Guangzhou, China

**Keywords:** elderly people, pneumonia, tradional medicine, chinese herbal injections, bayesian network meta-analysis

## Abstract

**Background:** Pneumonia is a prevalent and complicated disease among adults, elderly people in particular, and the debate on the optimal Chinese herbal injections (CHIs) is ongoing. Our objective is to investigate the comparative effectiveness of various CHIs strategies for elderly patients with pneumonia.

**Methods:** A comprehensive search strategy was executed to identify relevant randomized controlled trials (RCTs) by browsing through several databases from their inception to first, Feb 2020; All of the direct and indirect evidence included was rated by Network meta-analysis under a Bayesian framework.

**Results:** We ultimately identified 34 eligible randomized controlled trials that involved 3,111 elderly participants and investigated 4 CHIs combined with Western medicine (WM) (Xiyanping injection [XYP]+WM, Yanhuning injection [YHN]+WM, Tanreqing injection [TRQ]+WM, Reduning injection [RDN]+WM), contributing 34 direct comparisons between CHIs. Seen from the outcome of Clinical effective rate and time for defervescence, patients taking medicine added with CHIs [Clinical effective rate, XYP + WM(Odd ratio (OR): 0.74, 95%Credible intervals (CrIs):0.55–0.98), YHN + WM(OR: 0.66, 95%CrI: 0.45–0.95), TRQ + WM(OR: 0.65, 95%CrI: 0.50–0.83), RDN + WM(OR: 0.60, 95%CrI: 0.40–0.89); Time for defervescence, YHN + WM(Mean difference (MD): −2.11, 95%CrI: −3.26 to −0.98), XYP + WM(MD: −2.06, 95%CrI: −3.08 to −1.09), RDN + WM(MD: −1.97, 95%CrI: −3.61 to −0.35), TRQ + WM(MD: −1.69, 95%CrI: −2.27 to −1.04)] showed statistically better effect compared with participants in the Control group (CG) who only took WM. Meanwhile, based on the time for disappearance of cough, 3 out of 4 CHIs [TRQ + WM(MD: −2.56, 95%CrI: −3.38 to −1.54), YHN + WM(MD: −2.36, 95%CrI: −3.86 to −1.00) and XYP + WM(MD: −2.21, 95%CrI: −3.72 to −1.10)] strategies indicated improvement of clinical symptoms. Only XYP + WM(MD −1.78, 95%CrI: −3.29 to −0.27) and TRQ + WM (MD: −1.71, 95%CrI: −2.71 to −0.73) could significantly shorten the time for disappearance of pulmonary rales.

**Conclusion:** According to the statistical effect size (The surface under the cumulative ranking), we found that XYP + WM was presumably to be the preferable treatment for treating elderly patients with pneumonia compared with WM alone in terms of clinical effective rate. Our findings were based on very limited evidence and thus should be interpreted with caution. The application of the findings requires further research.

## Background

Pneumonia, a common disease with detrimental effect among adults worldwide, particularly elderly people, is posing substantial healthcare costs, and social burdens globally (GBD Diarrheal Diseases Collaborators, 2017). Study indicated that the number of old people aged over 80 hit the highest hospital admission rate record (16.4 cases/1,000 persons per year) for Community Acquired Pneumonia (CAP), a figure higher than that for common adults whose rate was 2.5 cases/1,000 people per year (Jain et al., 2015). To make matters worse, the global outbreak of coronavirus disease 2019, a relevant pneumonia, namely Severe Acute Respiratory Syndrome Coronavirus 2, is rapidly evolving and expanding on a global scale (Ronco et al., 2020). The ever-increasing cases of pneumonia imposes much stress on intensive care management and urges a systematical method to help policy-makers and clinicians in making the optimal decisions among numerous available therapies (Mandell et al., 2007). To further complicate the situation, despite the extensive use of drugs to treat the pneumonia patients, such as antiseptics, statins, as well as antibiotics, a first-tier preferred treatment was of huge benefit to patients’ health but it also brought about a great risk of drug resistance and various adverse drug reactions (ADRs) (Labeau et al., 2011; Zampieri et al., 2015). The number of people who were diagnosed with pneumonia was going upwards rather than downwards, which directly exacerbated both the financial and resources burden on healthcare system (GBD 2016 Causes of Death Collaborators, 2017).

In the past decade, owing to the unsatisfactory response to current treatments, a considerable amount of research have focused on Chinese herbal medicine (CHM) to address a series of clinical symptoms of pneumonia patients (Zhou et al., 2000). As a potent, selective and promising formulation, Chinese herbal injection (CHI) was obtained via extracting and purifying the effective and active herbs compounds. The CHIs have gained more popularity in China due to their instant effects, few side effects and remarkable tonic function, and their efficacy was supported by previous studies not only on respiratory system, but also on many other systems (Zhao et al., 2013; Li et al., 2016). However, up to now, evidence that proves the efficacy of CHIs treating pneumonia has remained scarce among older adults. There is no study comparing the efficacy of CHIs in elderly adults with pneumonia. The necessity to bridge this gap via Network meta-analysis (NMA) is as follows: CHI combined with Western medicine (WM) was considered as a comprehensive therapy that is relatively effective and safe for treating pneumonia. However, the conventional meta-analysis can merely group various CHIs into a comprehensive CHI and analyze them separately while NMA can evaluate each CHI individually and compare them simultaneously. Consequently, our study used Bayesian NMA method to ascertain exactly whether such CHIs are truly helpful in preventing pneumonia among elderly people and to rank the efficacy of them.

## Method

### Search Strategies

Based on the Pubmed, embase, Cochrane Central register of controlled trials, Wan Fang database, China National Knowledge Infrastructure database, Chinese Biomedical Literature database, Chinese Scientific Journal database, we adopted a comprehensive search strategy from their inception to first, Feb 2020 to identify the eligible randomized controlled trials (RCTs) which scrupulously evaluate the efficacy of CHIs among elderly people with pneumonias according to various proper diagnostic criteria.

Without any language restriction, the following Medical Subject Heading (MeSH) terms incorporating with keywords by using the framework of Boolean logical operators were adopted: “Pneumonias”, “Pneumonitis”, “Lung Inflammations”, “Chinese herbal”, “Chinese drugs”, “Chinese plant extracts”, “Traditional medicine”, “Randomized controlled trials”, additional text-terms as well. We additionally implemented a recursive manual-search to retrieve full-text studies from the bibliographies of obtained trails or the similar systematic reviews in order to check the potentially eligible studies we missed at first. Details of the search strategy was introduced in Supplementary File S1. At the same time, all the records were collected and processed in **Endnote X9** (Thompson ISI Research Soft, Philadelphia, PA) software.

### Inclusion and Exclusion Criteria

We defined the satisfactory randomized controlled trials based on the predetermined selection rules that met the following PICOS criterion:

**Population:** Pneumonitis was diagnosed by any proper clinical criteria, such as “Guidelines for diagnosis and treatment of community acquired pneumonia (Chinese revision)” (Academy RBoCM, 2006). Aged over 60 years old, without sex, race or region restriction.
**Intervention:** Based on the basis of literature and a pre-search we conducted before, patients with pneumonitis treated by numerous CHIs were conceptualized in four main CHIs, namely, Tanreqing injection (TRQ), Xiyanping injection (XYP), Yanhuning injection (YHN) and Reduning injection (RDN) (Ye, 2016; Xia, 2017; Yao, 2017; Zhou, 2017), while the other CHIs failed to report the sufficient available data for our analysis. We excluded the studies in which either different CHIs are combined or the definition of CHI is vague.
**Comparison:** CHIs or the control group alone. Placebo and most of them were WM.
**Outcomes:** Primary outcome: The clinical effective rate [CER, CER = (Total sample size of patients–total sample size of patients ineffective)/total sample size of patients * 100%]; Secondary outcomes: Clinical symptom disappearance time (cough disappearance, lung rale disappearance time).
**Study design:** We confined our study design to RCTs published without year and language restriction. Non-randomized controlled studies such as case-control studies, cohort studies, full-text but unpublished studies were all excluded.

Initially, two investigators respectively screened the collected studies by title and abstract. Duplications were removed primarily according to their titles and the first author’s name. Through a full-text review, all the potentially relevant articles were further perused and assessed at length. Our study adheres to the Network Meta-analysis of the Preferred Reporting Items for Systematic Reviews and Meta Analyses (PRISMA-NMA) (See in Supplementary File S2) and the Cochrane Handbook for Systematic Reviews of Interventions (Higgins and Green, 2011; Hutton et al., 2015). All analyses were based on previous published studies and therefore no ethical approval and patients’ consent were required.

### Data Extraction and Quality Assessment

Following the Cochrane consumers and Communication Review Group’s data extraction template, two investigators independently implemented a data extraction form to collect the relevant data of the included studies such as Essential publication information (name of first author, year of publication), characteristic of participants (Total sample size, baseline age), and quality of the RCT. Any conflict or discrepancy regarding screening reached consensus after discussion, or was judged by an expert as a third author.

The Cochrane Risk of Bias tool (ROB) was utilized to assess the quality of each trial, which divided ROB into seven items, and all trials were rated as low, unclear or high bias level (HGGS, 2011). For the selection bias, studies would be accepted as low risk of bias if they had described the details of sequence generation and approach of allocation concealment. Otherwise, the studies would be regarded as high risk of bias. We judged the items Performance and Detection Bias based on whether the study was blind or not to the participants, personnel and outcome assessors. For the attrition bias, we rated studies as high-risk whose data was insufficient for contributing to the analysis especially the primary outcome. For judging other biases, if the study had an imprecize study design, or reported an obvious inconsistency compared with the previous studies, they would be rated as high risk of bias. We assessed selective biases by detecting whether the included studies reported the raw data for analysis. All the 7 items were rated as “unclear risk” when the study did not mention the relevant items. We focused on the selection bias and attrition bias due to their specificity in the assessment of ROB. Any disagreement generated during the procession of assessment was resolved by team-discussion.

### Statistical Analyses

Our study first carried out a conventional pairwise meta-analysis to analyze the direct evidence by synthesizing the available data from the included studies. *I* ^*2*^ statistics were set to judge the magnitude of heterogeneity with higher values indicating more heterogeneity (Higgins and Thompson, 2002). The sensitivity analysis was conducted to test if there was any heterogeneity existing and to explore its sources. A network geometry was constructed firstly to provide comparative evidence among treatments, with distinguishing treatments expressed by different nodes whose size corresponded to the sample of participants, and studies were connected by appropriate lines. Moreover, in order to assess whether other publication biases or small-study biases were generated in our study, we created comparison-adjusted funnel plots to detect whether the scatter distributed to the vertical axis is asymmetric in the funnel plot (*X*-axis is sample size, *Y*-axis is effect) (Seagroatt and Stratton, 1998). The above analyses were conducted by using STATA/SE version 15.0 (Stata, corp, College Station, TX).

The Transitivity Assumption was applied to the appraisal of the robustness and reliability of the NMA by comparing the clinical similarity (e.g., patients, sample size and origin) and the methodological characteristics (e.g., random method, outcome and bias) among the studies involved (Caldwell et al., 2005). For outcomes presented as the numerical variable and the categorical variable, we generated a pooled Mean difference (MD) (Hedge’s *g*) and Odd ratio (OR) following 95% Credible intervals (CrIs) as a conservative estimate to summarize the treatment and effects respectively. Based on the maximum likelihood and Bayesian estimation, the approach of Markov-chain Monte Carlo with prior non-informative distributions were performed in our NMA (Salanti et al., 2008; Mavridis and Salanti, 2013). We set up three parallel chains running in our model simultaneously by means of Gibbs sampling and different initial values during the beginning period, through stimulation to obtain their target distributions (Valkenhoef and Kuiper, 2016). Each chain was put into 100,000 interactions and the first 20,000 were discarded to minimize the bias which may be produced at the initial stage. The Brooks-Gelman-Rubin diagnostic statistics were used to spot the convergence of model by checking the density plot and tract plot (Brooks and Gelman, 1998). The surface under the cumulative ranking (SUCRA), a concisely hierarchical curve, was produced to rank the possible efficacy of each treatment and it was found that higher SUCRA values denoted greater efficacy (Dias et al., 2012). Finally, the inconsistency of our NMA was examined by the split-node method and the *p*-value was obtained to indicate whether a significant inconsistency existed (Valkenhoef et al., 2016). All Bayesian analyses were done in R Version 3.32 (X64) with package “Gemtc”and WinBUGS Version1.4.3 (MRC Biostatistics Unit, Cambridge, United Kingdom) (Coreteam, 2014), and details of code were shown in Supplementary File S3.

## Results

### Study Selection

**FIGURE 1:**
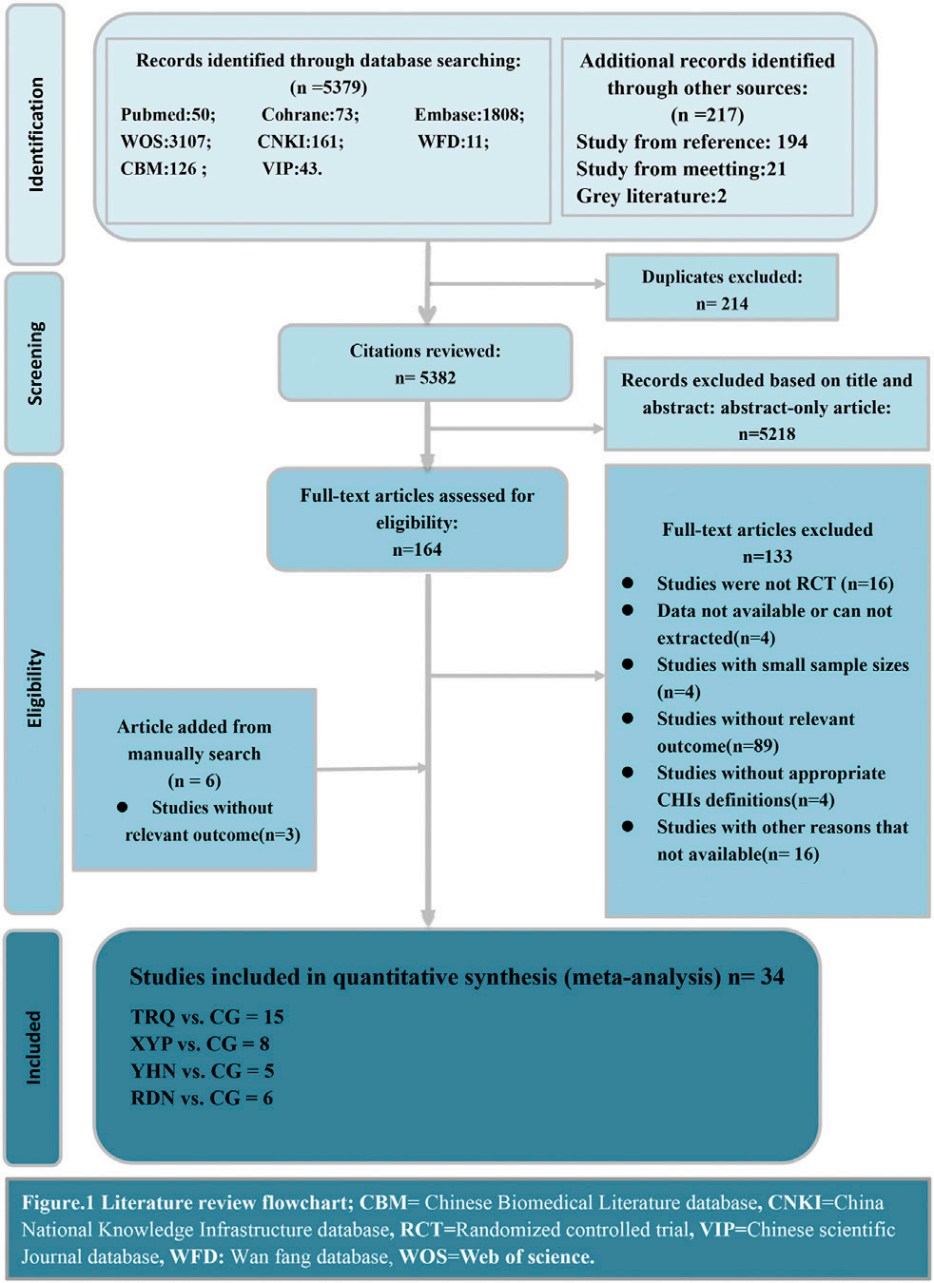
Literature review flowchart. CBM, Chinese Biomedical Literature database; CNKI, China National Knowledge Infrastructure database; RCT, Randomized controlled trial; VIP, Chinese scientific Journal database; WFD, Wan fang database; WOS, Web of science.

We obtained 5,596 records initially and reviewed 164 potentially relevant studies in full-text after screening the titles and abstracts. Overall, 214 nonduplicate citations were identified, and we retrieved 6 relevant articles by hand-search in case of omitting the eligible research we probably left out in the electronic database. 34 RCTs eventually met our pre-established criteria after a rigorous full-text review, which consisted of 4 CHIs treatments. The details of literature screen were depicted in Figure 1. All the investigators involved reached a unanimous approval for the selection and the assessment of the studies.

### Study Characteristics and Risk of Bias Quality Assessment

Supplementary Table S1 revealed the detailed demographic characteristics of 34 included studies. All the 34 parallel randomized controlled trials took place in China from 2008 to 2019. In total, 682 participants were randomly assigned to TRQ + WM group, 425 to XYP + WM, 213 to YHN + WM and 247 to RDN + WM whereas 1,544 were categorized into the CG (All the CG were conducted with WM only). Our NMA comprised 1,030 males participants and 2081 female participants, all of whom were qualified in terms of age according to the definition offered by World Health Organization (WHO) (WHO, 2017) that elderly people are those aged at least 60, The mean of all participants ranged from 60 to 85. Most trials (22/34, 64.71%) recruited participants with CAP, but few of them (4/34, 11.76%)considering the patients with Hospital Acquired Pneumonia (HAP). The citations of the included studies are introduced in detail in Supplementary File S4.

The overwhelming majority of the trials rendered a low risk of bias in “Random sequence generation” and all trials reflected a low risk of bias in Incomplete Outcome Data with sufficient original data. Notably, two studies were judged to be high-risk bias of “selective reporting” because their data for our analysis were calculated by transforming the original data manually (Li, 2010; Zhou, 2019). Due to their less rigorous study design, three studies (Huang, 2014; Zhang, 2014; Tian, 2016) were rated as high-risk bias in “other bias” items, among which the first two (Zhang, 2014; Tian, 2016) had less precise definition on pneumonia with one (Tian, 2016) not further reporting the calculation procedures for its relevant outcomes, and the third (Huang, 2014) had a bad representativeness of cases compared with other included studies since it recruited all the participants with pneumonia for a long-term period from 2000 to 2013. The remaining two items (“Allocation Concealment”, “Blinding of Participants and Personnel”) were generally ranked as unclear-to-moderate risk of bias. Full details regarding risk of bias for each RCT were shown in Supplementary Figure S1 and summarized in Supplementary Figure S2, respectively. Funnel plots throughout the included studies based on the four endpoints showed a relatively symmetric distribution (Supplementary Figure S3).

### Network Meta-analysis

**FIGURE 2:**
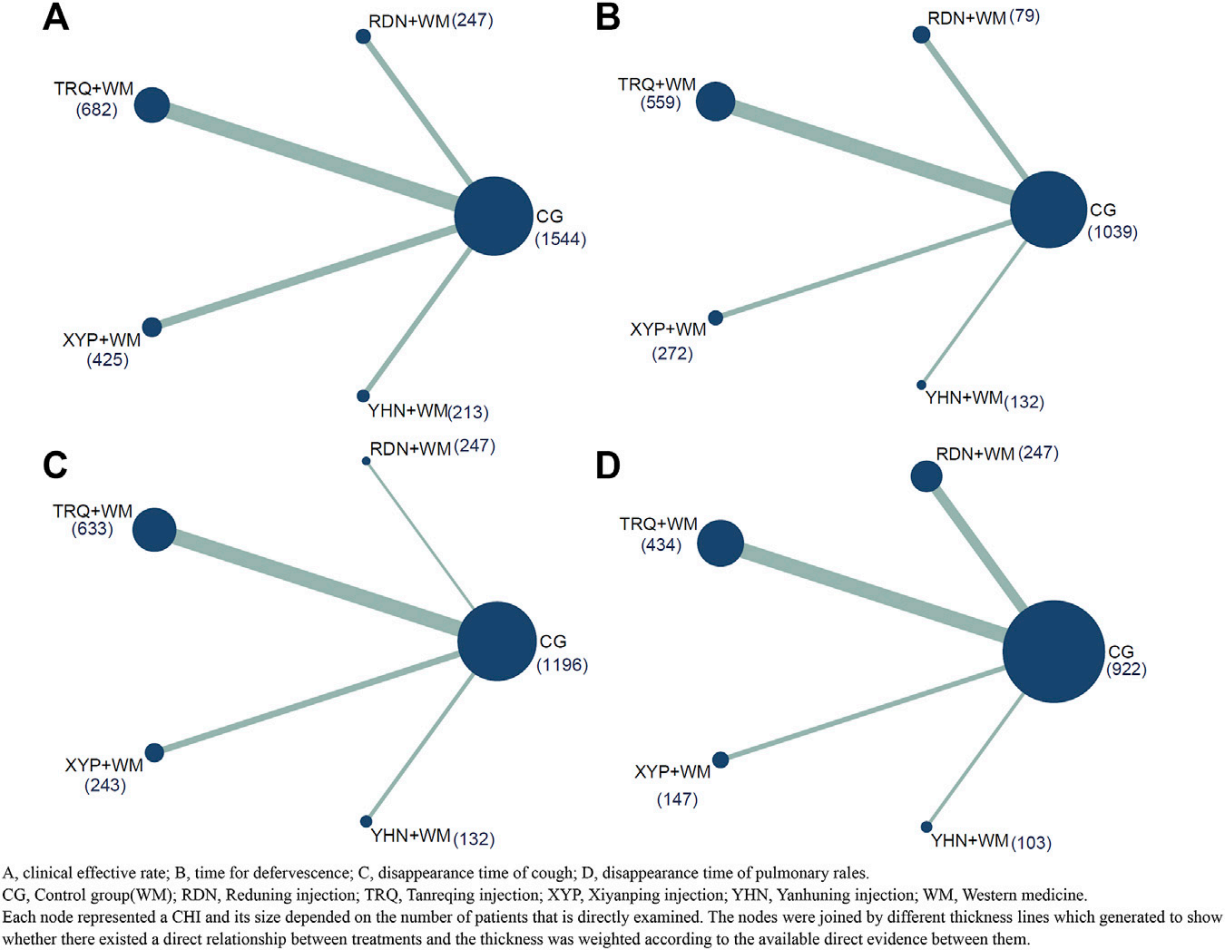
Network plot of all the trials based on the outcomes. **(A)** Clinical effective rate; **(B)** time for defervescence; **(C)** disappearance time of cough; **(D)** disappearance time of pulmonary rales. CG, Control group; RDN, Reduning injection; TRQ, Tanreqing injection; XYP, Xiyanping injection; YHN, Yanhuning injection; WM, Western medicine. Each node represented a CHI and its size depended on the number of patients that is directly examined. The nodes were joined by different thickness lines which generated to show whether there existed a direct relationship between treatments and the thickness was weighted according to the available direct evidence between them.

Figure 2 presented all the primary evidence between each CHI, and all the CHIs had at least one comparison with CG while all of CHIs lacked a closed loop between them. According to the network geometries of clinical effective rate, CG (1,544) obtained the largest sample size (SS) compared with other three CHIs whereas YHN + WM(213) got a relative lower SS throughout the entire network.

### Primary Outcome

#### Clinical Effective Rate

**FIGURE 3:**
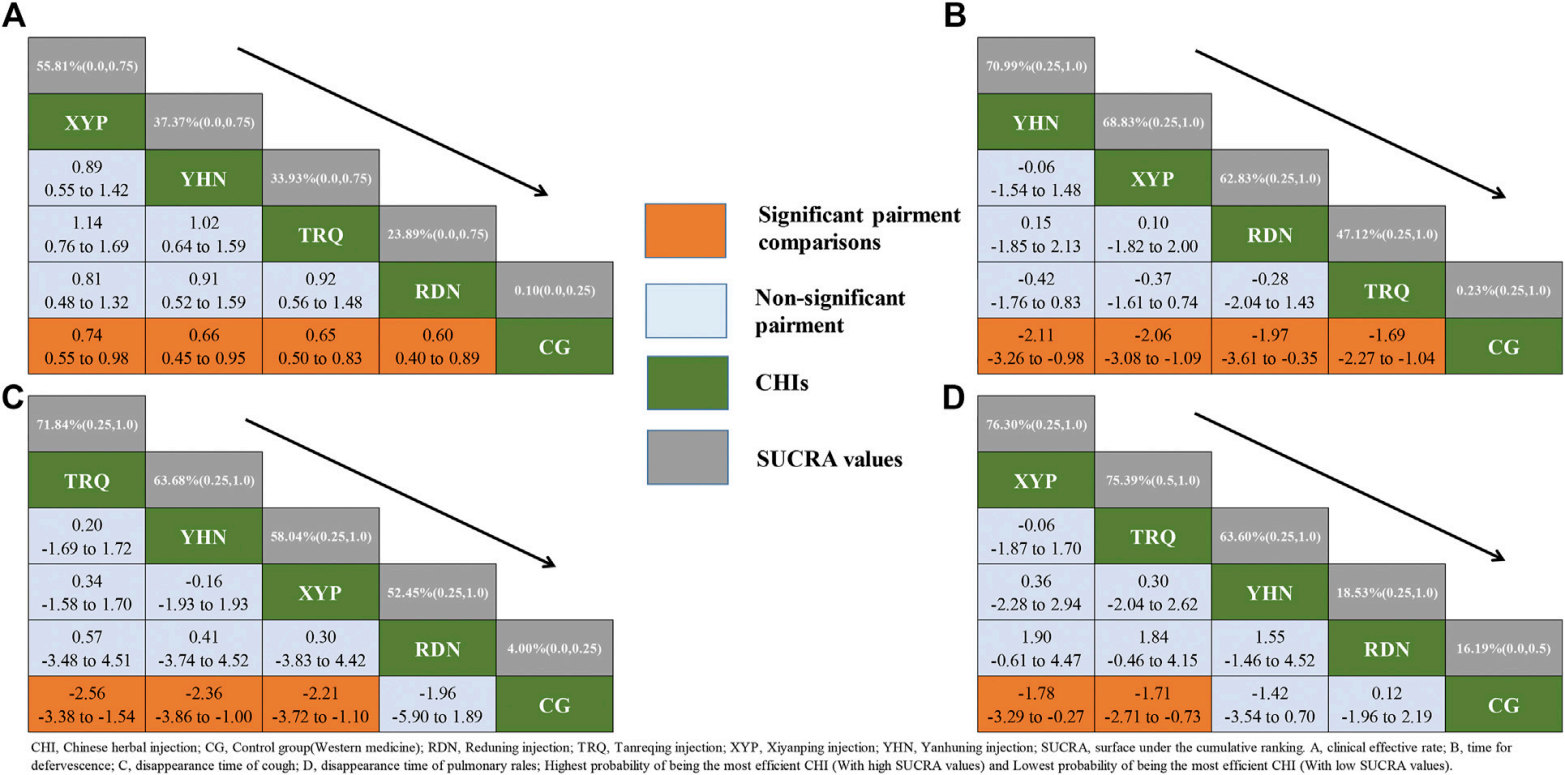
Relative effect sizes of efficacy at post-treatment according to network meta-analysis. CHI, Chinese herbal injection; CG, Control group (Western medicine); RDN, Reduning injection; TRQ, Tanreqing injection; XYP, Xiyanping injection; YHN, Yanhuning injection; SUCRA, surface under the cumulative ranking. **(A)** Clinical effective rate; **(B)** time for defervescence; **(C)** disappearance time of cough; **(D)** disappearance time of pulmonary rales; Highest probability of being the most efficient CHI(With high SUCRA values) and Lowest probability of being the most efficient CHI(With low SUCRA values).

In terms of the outcome of CER, all four CHIs had significant benefits to patients with pneumonia compared with CG. XYP + WM(OR:0.74, 95%CrI:0.55–0.98), YHN + WM(OR: 0.66, 95%CrI: 0.45–0.95), TRQ + WM(OR: 0.65, 95%CrI: 0.50–0.83), RDN + WM(OR: 0.60, 95%CrI: 0.40–0.89) (See in Supplementary Table S2). However, no significant difference was observed in any comparison among CHIs(See in Figure 3). Based on the SUCRA values, XYP + WM(SUCRA = 55.81%) took the lead in the effect on pneumonia patients in terms of CER compared with other three CHIs, closely followed by two equally remarkable CHIs, namely, YHN + WM(SUCRA = 37.37%) and TRQ + WM(SUCRA = 33.93%) while RDN + WM(SUCRA = 23.89%) lagged behind (See in Figure 3 and Supplementary Figure S4).

### Secondary Outcomes

#### The Time for Clinical Symptoms to Disappear

With regarding to the time for defervescence, all CHIs, similar to the above outcome, produced a significant efficacy compared with CG. YHN + WM(MD: −2.11, 95%CrI: −3.26 to −0.98), XYP + WM(MD: −2.06, 95%CrI: −3.08 to −1.09), RDN + WM(MD: −1.97, 95%CrI: −3.61 to −0.35), TRQ + WM(MD: −1.69, 95%CrI: −2.27 to −1.04) (See in Supplementary Table S2).

For cough disappearance, only TRQ + WM(MD: −2.56, 95%CrI: −3.38 to −1.54), YHN + WM(MD: −2.36, 95%CrI: −3.86 to −1.00) and XYP + WM(MD: −2.21, 95%CrI: −3.72 to −1.10) were associated with a significant improvement among patients. However, the results didn’t show statistical difference between RDN + WM (MD: −1.96, 95%CrI: −5.89 to 1.89) injection and other groups (See in Supplementary Table S2).

As for the time for disappearance of pulmonary rales, only XYP + WM(MD: −1.78, 95%CrI: −3.29 to −0.27) and TRQ + WM(MD: −1.71, 95%CrI: −2.71 to −0.73) had desirable performance in accelerating the disappearance of pulmonary rales (See in Supplementary Table S2).

Based on the SUCRA values, it seemed that among these CHIs injections, YHN + WM(SUCRA = 70.99%), TRQ + WM(SUCRA = 71.84%) and XYP + WM (SUCRA = 76.30%) became the optimal CHI compared to CG for the outcome of time for defervescence, cough disappearance and pulmonary rales disappearance, respectively. Conversely, TRQ + WM(SUCRA = 47.12%), RDN + WM(SUCRA = 52.45%) and RDN + WM(SUCRA = 18.53%) were most likely to be the least efficacious for defervescence, cough disappearance and pulmonary rales disappearance, respectively (See in Figure 3 and Supplementary Figure S4). According to the four endpoints, forest plot was conducted in order to display the efficacy of each CHI for improving the clinical symptoms among patients (Supplementary Table S2). Relative effect sizes of treatments efficacy according to all outcomes were shown in Figure 3.

## Discussion

This NMA has adopted a consecutive Bayesian model which involves 34 parallel randomized controlled trials to appraise 4 CHIs with four outcomes by comparing their effect among elderly patients with pneumonia. Considering the primary outcome, XYP + WM seemed to be most promising CHI in helping the elderly with pneumonia to improve their clinical symptoms whereas YHN + WM and TRQ + WM are suboptimal and can be regarded as complementary therapies.

Pneumonia represents a progressive and prevalent clinical condition due to its relatively high morbidity and mortality, and high incidence was found among elderly adults due to their higher susceptibility (Thomas et al., 2012). As the first-line preferred therapy which is widely used for respiratory disease, antibiotic is a double-edged sword, because though improving the clinical symptom of patients, it, as the same time, causes some adverse effects (AEs) (Mandell et al., 2007). Due to the fault of current treatment and much attention being concentrated on the efficacy of CHIs, our study was attempted to achieve relatively objective evidence in the hope of seeking the optimal CHI among elderly people in order to offer an evidence-base mirror.

From the perspective of Traditional Chinese Medicine (TCM), pneumonia belongs to the category of asthma and cough, and its clinical symptoms such as cough, expectoration, fever and asthma should be treated; Other symptoms caused by exogenous pathogens invading the lung should be focused on the way of allaying fever and soothing the lung (Feng, 2014). Modern pharmacology shows that CHIs have multiple effects on antipyretic and analgesic, antiviral and antibacterial symptoms (Li et al., 2017), such as XYP injection. As a derivative of andrographolid with the characteristics of antipyretic and anti-inflammatory, it holds the most likelihood to be the optimal CHI injection among 4 CHIs, which enhances the immunity of the body. Moreover, XYP can improve the smooth muscle spasm, relax the smooth muscle, control the symptoms of cough and resolve phlegm by reducing the recreation of serous fluid (Zhang, 2011; L, 2017). TRQ classifies Chinese herbal into five categories: Scutellaria, Bear(bile) powder, Cornu gorais, honeysuckle and forsythia, with the efficacy of heat-clearing and detoxicating. It also has a positive effect on fever, cough and sore throat triggered by pulmonary disease (Huang, 2018). Patients with pneumonia would ameliorate their symptom by the usage of YHN injection or RDN injection, which was corroborated by numerous studies. But the two aforementioned CHIs got a relatively poor ranking in our study, possibly due to that they two mainly played a role in inhibiting respiratory viruses and blocking bacterial growth among patients with viral pneumonia rather than with the ability of anti-inflammatory, antipyretic and immunoregulatory effects among CAP patients (Gao et al., 2011; Nie, 2018). Although conclusions drawn by numerous studies showed consistency with our study that most such CHIs were preferable in the clinical treatment for patients with pneumonia when compared with CG (Sun, 2019; Wang, 2018). But there is no significant difference detected in any comparison between CHIs due to the absence of the indirect evidence among them. XYP + WM seem to be the most effective CHI for the outcome of clinical effective rate and the disappearance time of pulmonary rales while based on the results of time for defervescence and the disappearance time of cough that YHN + WM and TRQ + WM got the highest possibility become the most effective for improving these clinical symptoms, respectively. In spite of the evidence regarding the effectiveness of YHN + WM on reducing the fever is limited, some relatively convincing evidence can account for it that YHN + WM is an effective extract of Andrographis paniculate which was recommended by Chinese medicine as one of the most effective herbal with the function of clearing away heat and toxic material (Tian, 2011). Compared with other CHIs, the main function of TRQ is diluting the sputum which makes the sputum thinner and easier to cough up and further improves the patients’ breath, so that the clinical symptoms of cough can disappear in a short time (Ye, 2016). One previous study has compared the effect of various CHIs on CAP for all age groups and our study only shared similar analysis methods with it. Focusing on elderly people and all the types of pneumonia, our study arrived at a conclusion that XYP + WM might be the best CHI for improving elderly people with pneumonia, which was inconsistent with the results of the previous study that YHN + WM was the best CHI for treating pneumonia (Huang et al., 2019). The difference of research subjects may account for the different conclusions between our study and the former one since our study was more specifically targeted at elderly patients rather than patients of all ages. As we mentioned before, although YHN had rapid onset of action, as well as good bioavailability widely used in clinical, but it had a greater incidence of toxicities or AEs (Nie, 2018). One study indicated that YHN injection had the highest incidence of AEs among numerous CHIs (Xiong et al., 2016), and the Food and Drug Administration of China also indicated the usage of YHN was accompanied with substantial AEs especially the systemic damage (China Tfadao, n d). The above findings would diminish metabolizing capacity and excretory functions in the elderly patients since metabolism and excretion are significant parameters to evaluate the transformation and delivery of drugs in the body (Gao, 2017; Gao et al., 2017). As a result, it was more likely to lead to accumulation of drugs in elderly bodies and increased their risk of AEs compared with ordinary adults, and thus decreased the effectiveness of TCM for elderly people in terms of population (Gao, 2017). We may hypothesize that elderly population was probably sensitive and fragile to be affected by the acceptability of TCM especially the drug toxicology of TCMs. Therefore, the XYP + WM is the most effective based on the result of our study while the previous study focus on ordinary adults is in favor of YHN + WM.

### Strength and Limitations

Instead of performing a traditional meta-analysis, our study lies in the fact that we structured a Bayesian framework to evaluate the effectiveness of current CHIs simultaneously for pneumonia patients among elderly population based on several endpoints. Moreover, we enriched the way of search strategy in order that more potentially eligible trials would be found for the establishment of the literature basis of our NMA.

The limitations across our study also should not be ignored. First, we have not registered our study either in Cochrane central or PROSPERO, which may indirectly influence the generalization of our results. Due to the fact that the topic of our study is CHI, all trials were simply conducted in China and our NMA therefore might not apply to cases in other countries. We have not collected and analyzed the data regarding AEs, because only two studies mentioned the AEs(One study conducted with XYP + WM found a patient getting a rash during the therapy duration while the other study reported several patients with bloating, nausea, vomiting and facial flushing after taking YHN + WM) and we could not estimate it without sufficient data (Yuan, 2010; Wang, 2014). Quality of several studies would possibly weaken the reliability and robustness of our results. Only two studies mentioned the blind method (Zhang, 2010; Guo, 2019a), and two studies were rated as low risk of bias in selection bias (Li, 2018; Guo, 2019b). Moreover, there were three trials with a high risk of bias with regard to other biases (Huang, 2014; Zhang, 2014; Tian, 2016).

## Conclusion

In summary, based on the clinical effective rate, our NMA suggested that XYP + WM probably was the better CHI choice among numerous CHIs for elderly patients with pneumonia when compared to WM. Meanwhile, it worth mentioning that this finding should be interpreted with caution due to the limited evidence. Besides, the present study put emphasis on the need of future guidelines to optimize their management and further similar high-quality trials should be carried out to confirm our results.

## Data Availability

The original contributions presented in the study are included in the article/Supplementary Material, further inquiries can be directed to the corresponding authors.
