# Supplementary material for: Efficacy of Chinese Herbal Injections for Elderly Patients With pneumonia—A Bayesian Network Meta-analysis of Randomized Control Trials
Source: Front Pharmacol. 2021 May 21;12:610745. doi: 10.3389/fphar.2021.610745 (PMC8176116; doi:10.3389/fphar.2021.610745)
Supplement: Supplementary file 1 [file Table1.pdf]

Supplement Details respecting to the information of 4 CHIs

Injection name	Source	Species/Raw materials	Component ingredients to be measured	Botancical plant names	Quality control reported? (Y/N)	Chemical analysis reported? (Y/N)
Xiyanping injection	Jiangxi Qingfeng Pharmaceutical Co., Ltd.	Andrographolide sulfonate	Andrographolide sulfonate, 90.0%-110.0% of labelled amount	Andrographis paniculata (Burm.f.) Nees	Y-WS-10863 (ZD-0863) -2002-2011Z issued by National Food and Drug Administration National Drug Standards. Standard number	N
Yanhuning injection	Hainan Tongyongkangli Pharmaceutical Co., Ltd;<br>Guangdong Xianqiang Pharmaceutical Co., Ltd;<br>Hainan Star Pharmaceutical Co., Ltd;<br>Chongqing Lummy Pharmaceutical Co.,Ltd;<br>Haikou Qili Pharmaceutical Co., Ltd;<br>Fujian Mindong Rejuvenation Pharmaceutic al Co., Ltd;<br>Harbin Wandashan pharmaceutical Co., Ltd.	Potassium Sodium Dehydroandro and rographolide Succinate	Potassium Sodium Dehydroandroand rographolide Succinate, 90.0%-110.0% of labelled amount	Andrographis paniculata (Burm.f.) Nees	Y-YBH06272009 issued by National Food and Drug Administration National Drug Standards. Standard number	N
Tanreqing injection	Shanghai Kaibao Pharmaceutical Co., Ltd.	Scutellariae Radix,<br>Bear bile powder,<br>Cornu gorais,<br>Lonicerae Japonicae Flos,<br>Forsythiae Fructus	Baicalin, › 0.5mg/mL; ursodeoxycholic Acid, › 5.4mg/mL; alanine, › 1.75-3.005.4mg/mL	Scutellaria baicalensis Georgi,<br>Selenarctos thibetanus Cuvier,<br>Capra hircus Linnaeus,<br>Lonicera japonica Thunb,<br>Forsythia suspensa (Thunb.) Vahl	Y-YBZ00912003-2007Z-2009-2012 issued by National Food and Drug Administration National Drug Standards. Standard number	N
Reduning injection	Jiangsu Kanion Pharmaceutical Co., Ltd.	Artemisiae Annuae Herba,<br>Lonicerae Japonicae Flos,<br>Gardznize Fructus	Gardenoside, 9.0-14.0mg/mL; chlorogenic acid, 5.6-8.4mg/mL	Artemisia annua L.,<br>Lonicera japonica Thunb,<br>Gardenia jasminoides J.Ellis	Y-YBZ08202005 issued by National Food and Drug Administration National Drug Standards. Standard number	N
