# Supplementary material for: Efficacy of Chinese Herbal Injections for Elderly Patients With pneumonia—A Bayesian Network Meta-analysis of Randomized Control Trials
Source: Front Pharmacol. 2021 May 21;12:610745. doi: 10.3389/fphar.2021.610745 (PMC8176116; doi:10.3389/fphar.2021.610745)
Supplement: Supplementary file 2 [file Image4.pdf]

Supplement Figure.4 SUCRA plot for four outcomes

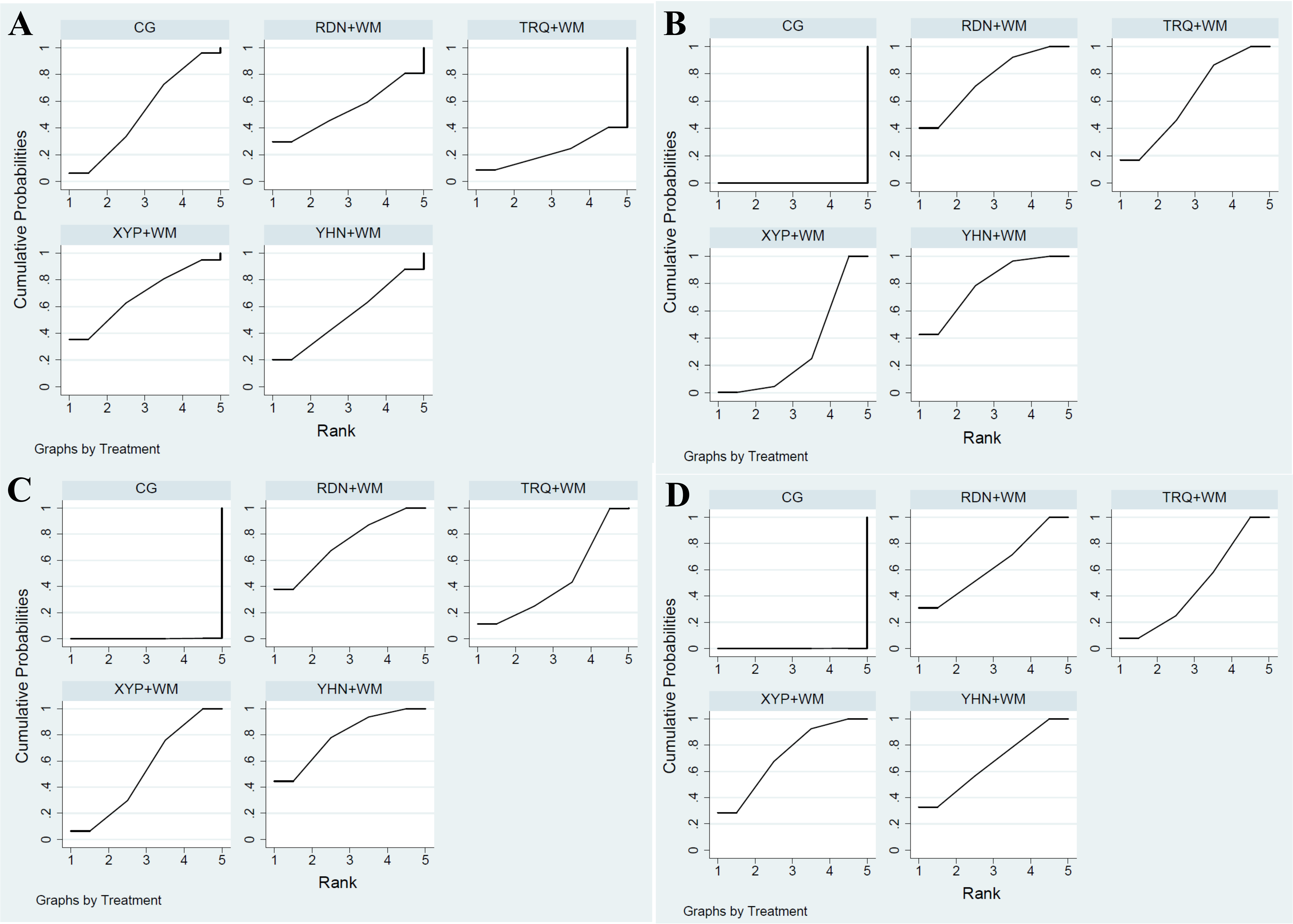

A, clinical effective rate; B, time for defervescence; C, disappearance time of cough; D, disappearance time of pulmonary rales.
CG, Control group; RDN, Reduning injection; TRQ, Tanreqing injection; XYP, Xiyanping injection; YHN, Yanhuning injection; WM, Western medicine.
Highest probability of being the most efficient CHI(With high SUCRA values) and Lowest probability of being the most efficient CHI(With low SUCRA values).
