# Supplementary material for: Efficacy of Chinese Herbal Injections for Elderly Patients With pneumonia—A Bayesian Network Meta-analysis of Randomized Control Trials
Source: Front Pharmacol. 2021 May 21;12:610745. doi: 10.3389/fphar.2021.610745 (PMC8176116; doi:10.3389/fphar.2021.610745)
Supplement: Supplementary file 4 [file Image3.pdf]

Supplement Figure.3 Funnel plot

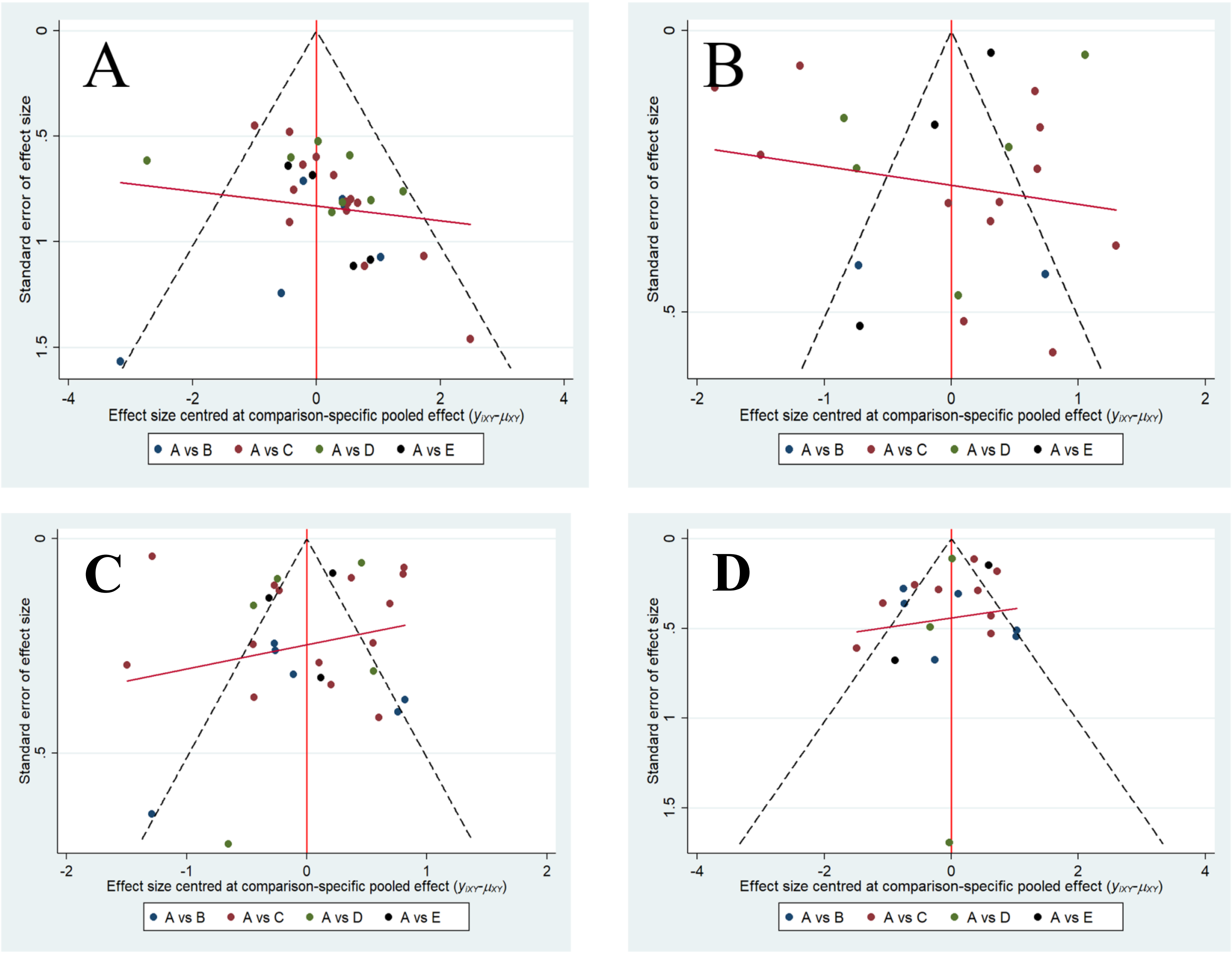

A vs B, CG vs TRQ+WM; A vs C, CG vs XYP+WM; A vs D, CG vs THN+WM; A vs E, CG vs RDN+WM.
A, clinical effective rate; B, time for defervescence; C, disappearance time of cough; D, disappearance time of pulmonary rales.
