# Supplementary material for: Efficacy of Chinese Herbal Injections for Elderly Patients With pneumonia—A Bayesian Network Meta-analysis of Randomized Control Trials
Source: Front Pharmacol. 2021 May 21;12:610745. doi: 10.3389/fphar.2021.610745 (PMC8176116; doi:10.3389/fphar.2021.610745)
Supplement: Supplementary file 5 [file DataSheet1.pdf]

```
model{ # *** PROGRAM STARTS
for(i in 1:ns){ # LOOP THROUGH STUDIES
w[i,1] <- 0 # adjustment for multi-arm trials is zero for control arm
delta[i,1] <- 0 # treatment effect is zero for control arm
mu[i] ~ dnorm(0,.0001) # vague priors for all trial baselines
for (k in 1:na[i]) { # LOOP THROUGH ARMS
var[i,k] <- pow(se[i,k],2) # calculate variances
prec[i,k] <- 1/var[i,k] # set precisions
y[i,k] ~ dnorm(theta[i,k],prec[i,k]) # normal likelihood
theta[i,k] <- mu[i] + delta[i,k] # model for linear predictor
dev[i,k] <- (y[i,k]-theta[i,k])*(y[i,k]-theta[i,k])*prec[i,k] #Deviance contribution
}
resdev[i] <- sum(dev[i,1:na[i]]) # summed residual deviance contribution for this trial
for (k in 2:na[i]) { # LOOP THROUGH ARMS
delta[i,k] ~ dnorm(md[i,k],taud[i,k]) # trial-specific LOR distributions
md[i,k] <- d[t[i,k]] - d[t[i,1]] + sw[i,k] # mean of treat effects distributions (with multi-
arm trial correction)
taud[i,k] <- tau *2*(k-1)/k # precision of treat effects distributions (with multi-arm trial
correction)
w[i,k] <- (delta[i,k] - d[t[i,k]] + d[t[i,1]]) # adjustment for multi-arm RCTs
sw[i,k] <- sum(w[i,1:k-1])/(k-1) # cumulative adjustment for multi-arm trials
}
}
totresdev <- sum(resdev[]) #Total Residual Deviance
d[1]<-0 # treatment effect is zero for reference treatment
for (k in 2:nt){ d[k] ~ dnorm(0,.0001) } # vague priors for treatment effects
sd ~ dunif(0,5) # vague prior for between-trial SD.
tau <- pow(sd,-2) # between-trial precision = (1/between-trial variance)
# Ranking and probabilities for treatment
for(k in 1:nt) {
order[k]<- nt+1-rank(d[],k)    #events are good
most.effective[k]<-equals(order[k],1)
for(j in 1:nt) {
effectiveness[k,j]<- equals(order[k],j)
cumeffectiveness[k,j]<- sum(effectiveness[k,1:j])
}
}
#SUCRA
for(k in 1:nt) {
SUCRA[k]<- sum(cumeffectiveness[k,1:(nt-1)]) /(nt-1)
}
# all MDs for each treatment level comparison
for (c in 1:(nt-1)) {
for (k in (c+1):nt) {MD[c,k] <- (d[k]-d[c]) } }}
```
