# Supplementary figures and images for: Efficacy of Chinese Herbal Injections for Elderly Patients With pneumonia—A Bayesian Network Meta-analysis of Randomized Control Trials
Source: Front Pharmacol. 2021 May 21;12:610745. doi: 10.3389/fphar.2021.610745 (PMC8176116; doi:10.3389/fphar.2021.610745)

Supplement Figure.1 Risk of Bias graph

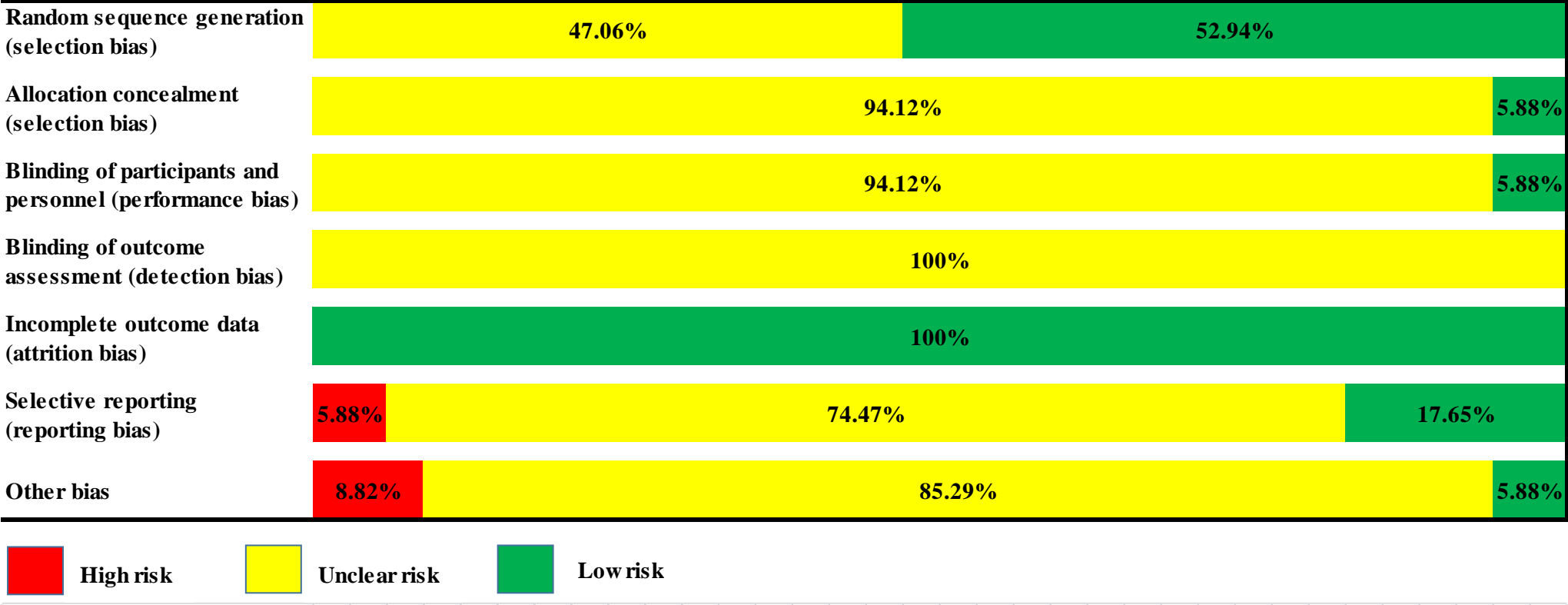

Supplement: Supplementary file 7 [file Image1.pdf]
