# Supplementary material for: Efficacy of Chinese Herbal Injections for Elderly Patients With pneumonia—A Bayesian Network Meta-analysis of Randomized Control Trials
Source: Front Pharmacol. 2021 May 21;12:610745. doi: 10.3389/fphar.2021.610745 (PMC8176116; doi:10.3389/fphar.2021.610745)
Supplement: Supplementary file 3 [file Image2.pdf]

Supplement Figure.2 Risk of Bias Summary

- High risk
- Unclear risk
- Low risk

	Bin Zhang, 2010	Qiuxi Liang, 2015	Min Ye, 2016	Xue lin Zhang, 2016	Honghui Chen, 2017	Fang Tao, 2017	Yanling Wang, 2017	Tingting Liu, 2019	Zhiyan Liu, 2012	Zhimin Zhang, 2014	Jianwei Hu, 2015	Qiang Hu 2008	Yumei Guo, 2019	Yanhui Sun, 2017	Hongwei Chen, 2019	Jing Li, 2010	Jinghua Liu, 2013
Random sequence generation (selection bias)	Unclear	Unclear	Low	Unclear	Low	Unclear	Low	Low	Unclear	Unclear	Low	Unclear	Low	Unclear	Unclear	Unclear	Unclear
Allocation concealment (selection bias)	Unclear	Unclear	Unclear	Unclear	Unclear	Unclear	Unclear	Unclear	Unclear	Unclear	Unclear	Unclear	Unclear	Unclear	Unclear	Unclear	Unclear
Blinding of participants and personnel (performance bias)	Low	Unclear	Unclear	Unclear	Unclear	Unclear	Unclear	Unclear	Unclear	Unclear	Unclear	Unclear	Low	Unclear	Unclear	Unclear	Unclear
Blinding of outcome assessment (detection bias)	Unclear	Unclear	Unclear	Unclear	Unclear	Unclear	Unclear	Unclear	Unclear	Unclear	Unclear	Unclear	Unclear	Unclear	Unclear	Unclear	Unclear
Incomplete outcome data (attrition bias)	Low	Low	Low	Low	Low	Low	Low	Low	Low	Low	Low	Low	Low	Low	Low	Low	Low
Selective reporting (reporting bias)	Unclear	Unclear	Low	Low	Low	Unclear	Unclear	Low	Unclear	Unclear	Unclear	Unclear	Low	Unclear	Unclear	High	Unclear
Other bias	Unclear	Unclear	Unclear	Unclear	Unclear	Unclear	Unclear	Unclear	Unclear	Unclear	Unclear	Unclear	Unclear	Unclear	Unclear	Unclear	Unclear

	Jisheng Yuan, 2010	Jielin Guo, 2019	Yuheng Tian,2016	Hajimuhan Payiza,2015	Zhiqiang Yao	Lili Zhang, 2015	Wenli Huang, 2014	Jianhua Tian, 2011	Rong Wang, 2014	Yutao Zhou, 2019	Leifeng Xia, 2017	Zhaoqun Li, 2011	Fang Zhang, 2014	Minjie Wu, 2013	Jing Zhou, 2017	Dongmei Wang, 2018	Ping Li, 2018
Random sequence generation (selection bias)	Unclear	Low	Unclear	Low	Low	Low	Low	Low	Unclear	Unclear	Low	Unclear	Low	Low	Low	Low	Low
Allocation concealment (selection bias)	Unclear	Low	Unclear	Unclear	Unclear	Unclear	Unclear	Unclear	Unclear	Unclear	Unclear	Unclear	Unclear	Unclear	Unclear	Unclear	Low
Blinding of participants and personnel (performance bias)	Unclear	Unclear	Unclear	Unclear	Unclear	Unclear	Unclear	Unclear	Unclear	Unclear	Unclear	Unclear	Unclear	Unclear	Unclear	Unclear	Unclear
Blinding of outcome assessment (detection bias)	Unclear	Unclear	Unclear	Unclear	Unclear	Unclear	Unclear	Unclear	Unclear	Unclear	Unclear	Unclear	Unclear	Unclear	Unclear	Unclear	Unclear
Incomplete outcome data (attrition bias)	Low	Low	Low	Low	Low	Low	Low	Low	Low	Low	Low	Low	Low	Low	Low	Low	Low
Selective reporting (reporting bias)	Unclear	Low	Unclear	Unclear	Unclear	Unclear	Unclear	Unclear	Unclear	High	Unclear	Unclear	Unclear	Unclear	Unclear	Unclear	Unclear
Other bias	Unclear	Low	High	Unclear	Unclear	Unclear	High	Unclear	Unclear	Unclear	Unclear	Unclear	High	Unclear	Unclear	Unclear	Low
